# The importance of three dimensional coronary artery reconstruction accuracy when computing virtual fractional flow reserve from invasive angiography

**DOI:** 10.1038/s41598-021-99065-7

**Published:** 2021-10-04

**Authors:** Roshni Solanki, Rebecca Gosling, Vignesh Rammohan, Giulia Pederzani, Pankaj Garg, James Heppenstall, D. Rodney Hose, Patricia V. Lawford, Andrew J. Narracott, John Fenner, Julian P. Gunn, Paul D. Morris

**Affiliations:** 1 Department of Infection, Immunity and Cardiovascular Disease, The Medical School, University of Sheffield, Sheffield, UK; 2 Department of Cardiology, Sheffield Teaching Hospitals NHS Foundation Trust, Sheffield, UK; 3 Insigneo Institute for In Silico Medicine, University of Sheffield, Sheffield, UK; 4 Department of Radiology, Sheffield Teaching Hospitals NHS Foundation Trust, Sheffield, UK; 5 Norwich Medical School, University of East Anglia, Norwich, UK

**Keywords:** Translational research, Interventional cardiology

## Abstract

Three dimensional (3D) coronary anatomy, reconstructed from coronary angiography (CA), is now being used as the basis to compute ‘virtual’ fractional flow reserve (vFFR), and thereby guide treatment decisions in patients with coronary artery disease (CAD). Reconstruction accuracy is therefore important. Yet the methods required remain poorly validated. Furthermore, the magnitude of vFFR error arising from reconstruction is unkown. We aimed to validate a method for 3D CA reconstruction and determine the effect this had upon the accuracy of vFFR. Clinically realistic coronary phantom models were created comprosing seven standard stenoses in aluminium and 15 patient-based 3D-printed, imaged with CA, three times, according to standard clinical protocols, yielding 66 datasets. Each was reconstructed using epipolar line projection and intersection. All reconstructions were compared against the real phantom models in terms of minimal lumen diameter, centreline and surface similarity. 3D-printed reconstructions (n = 45) and the reference files from which they were printed underwent vFFR computation, and the results were compared. The average error in reconstructing minimum lumen diameter (MLD) was 0.05 (± 0.03 mm) which was < 1% (95% CI 0.13–1.61%) compared with caliper measurement. Overall surface similarity was excellent (Hausdorff distance 0.65 mm). Errors in 3D CA reconstruction accounted for an error in vFFR of ± 0.06 (Bland Altman 95% limits of agreement). Errors arising from the epipolar line projection method used to reconstruct 3D coronary anatomy from CA are small but contribute to clinically relevant errors when used to compute vFFR.

## Introduction

Invasive coronary angiography (CA) remains the standard method for assessing coronary artery disease (CAD) and guiding its treatment with percutaneous coronary intervention (PCI). Standard CA provides a two-dimensional (2D), dynamic, arterial ‘luminogram’ which is interpreted visually by the operator. By applying mathematical algorithms, 3D coronary anatomy can now be reconstructed using computer software from orthogonal 2D projections. 3D CA reconstructions more accurately capture lesion length, plaque eccentricity and correlate better with functional measures of disease, which is frequently overestimated using 2D QCA^1–3^. Moreover, by applying the governing equations of fluid dynamics to these reconstructions, physiological parameters such as pressure and flow can be predicted^4^.

3D CA reconstructions are now being used as the basis for the computation of clinical indices of coronary physiology such as fractional flow reserve (FFR). This is a significant development, because FFR improves patient outcomes and is considered the gold standard method for selecting appropriate cases for PCI in international guidelines^5,6^. The ability to compute FFR using 3D reconstructions from CA, known as angiography-derived or ‘virtual’ FFR (vFFR), is anticipated to widen access to the benefits physiologically-guided intervention, allowing the clinical benefits of FFR to become available to patients without the need to induce hyperaemia or pass an invasive pressure wire^7^. It is therefore, important that vFFR is accurate. Since the original VIRTUheart™ method of vFFR (University of Sheffield)^8^ several other methods have been developed and are now available commercially including quantitative flow reserve (QFR, MedisMedical Imaging), FFRangio (CathWorks) and vFFR (CAAS, Siemens Heathcare)^3,9,10^.

Compared with invasively measured FFR, vFFR has a typical error range (95% CI) of FFR ± 0.14^11^. vFFR errors arise from inaccuracies in the 3D CA reconstruction and in the assumptions and simplifications in the calculation of pressure gradients^7^. Despite the importance of accurate 3D CA reconstruction in applications used to compute vFFR, there is a paucity of published data validating their accuracy. Furthermore, the amount of vFFR error attributable to the reconstruction method is unknown.

The aims of this study were to:

1. Validate the geometric accuracy of a method for reconstructing 3D coronary artery anatomy from standard CA.
2. Quantify how much vFFR error results from errors in the reconstruction method.

## Methods

### Study design

**Figure 1:**
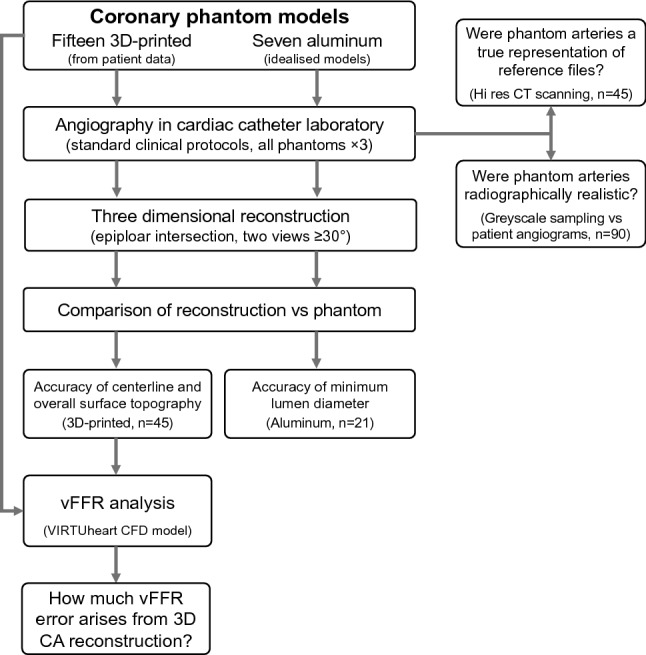
A flowchart demonstrating the experimental phantom arterial models, clinical imaging, 3D reconstruction, validation, vFFR simulation and analysis.

The study was performed at the University of Sheffield, and the South Yorkshire Cardiothoracic Centre at Sheffield Teaching Hospitals (STH) NHS Foundation Trust, and was approved by the regional institutional review board. The study was conducted according to all relevant research and development guidelines and regulations. Participating patients provided informed consent. First, we developed a method of 3D CA reconstruction. Next, the accuracy of this reconstruction method was validated using phantom arterial models which were imaged with CA according to standard clinical imaging protocols and then reconstructed. The geometry (surface similarity, centreline and diameter) of the reconstruction was then compared with the known geometry of the phantom models. This step validated the *geometric* accuracy of the reconstruction method. We then applied computational fluid dynamics (CFD) modelling to the reference files (the files from which the phantoms were 3D printed), and to the reconstructed arteries, to predict vFFR. By comparing the vFFR results, we were able to quantify the amount of vFFR error attributable directly to the reconstruction method. A summary flowchart illustrating the study design is shown in Fig. 1. A detailed explanation follows below**.**

### Reconstructing 3D coronary anatomy from coronary angiography

Two angiographic projections were selected, ≥ 30° apart, ensuring optimal visualisation of the artery and stenosis, during end diastole, as indicated by the software’s inbuilt ECG gating. Image selection does have an impact on vFFR results and a protocol for clincal imaging and appropriate image selection were published recently^4,12^. The arterial centrelines in each of two angiographic projections were computed automatically. The centreline in the first projection was discretised into a series of points and the epipolar lines of each point in the second projection were computed. Each corresponding point in the second projection was computed at the intersection of the epipolar line with the centreline in the second projection. The three-dimensional co-ordinates of each point were then computed from the corresponding projected co-ordinates. The diameter of the vessel in each projection was computed based upon automatic detection of the constrast gradient with manual correction where necessary (overlapping vessels or branch points). 3D luminal reconstruction assumed an axisymmetric vessel with the average diameter from the two projections. In the catheterisation laboratory it is normal to re-centre the region of interest by movement of the table between projections. Tracking of these movements is not universal, and there are other sources of motion, including patient cardiac and respiratory movement. Three points of correspondence in each of the two projections (usually bifurcations) were identified. Using these points and a linear, least-squares optimisation process, table and other movements were compensated for. The epipolar lines in the second projection were then computed, incorporating the computed table shift, and the reconstruction proceeded as before. The surfaces of the arteries were computed and represented as an assembly of triangular elements in STL format.

### Creating the coronary phantom models

Two types of arterial phantom models were created. To assess the accuracy of the method in reconstructing vessel diameter, particularly in the stenosis region (minimal lumen diameter), phantom models were created from 3.2 mm diameter aluminium rods, which were curved to mimic the contour of the epicardial surface. Both concentric and eccentric stenoses were fabricated by hand. Seven fabricated stenoses represented anatomically mild, moderate and severe disease, ranging from 44.7 to 77.2% diameter stenoses (Supplementary Table S1). The aluminium rods were chosen because, unlike the 3D printed models, they were strong enough to maintain shape and integrity even with severe stenoses. Diameter measurements were made with a high-precision digital caliper (Mitutoyo, KA, Japan) as the average of three readings. The second type of phantoms were 3D-printed using as their basis, the CAs of patients with chronic coronary syndromes in whom FFR had also been measured. These solid phantom models were used to assess the accuracy of the reconstruction’s overall surface topography. These were based on the angiograms of patients with chronic coronary syndromes, mean age 66 years, who were being assessed for PCI with FFR guidance. Three left and three right branched coronary arterial (LCA, RCA) models were printed using stereo lithography (Rep Rap X400 PRO 3D printer) in polylactic acid (PLA) doped with stainless steel to mimic the radiodensity of contrast-filled vessels under CA imaging. The six models comprised a total of 15 individual branches, which were analysed separately, as they normally would be clinically and physiologically.

### Angiographic imaging

All phantom models underwent routine coronary angiography (multiplane 2D acquisitions, Philips Azurion, Philips Healthcare, NL), according to a standard clinical protocol. Each phantom model was elevated to the height of a patient’s heart and positioned and held in anatomical orientation on the cath lab table. A consultant cardiologist and superintendent radiographer were instructed to image the model as if performing a clinical case, ensuring table movement and panning, in x, y and z planes. Projection angles for RCAs included LAO, RAO and LAO-cranial. Projection angles for LCAs included LAO-caudal, PA-caudal, RAO-caudal, LAO-cranial, PA-cranial and RAO-cranial (Supplementary Table S2). Images were exported in Digital Imaging and Communications in Medicine (DICOM) format. Each phantom model underwent three separate angiogram studies. The pixel:mm ratio for the Philips angiography system was 1 pixel = 0.314 mm.

### Ensuring the phantom models were radiographically realistic

To ensure the phantom models were radiographically realistic under angiographic imaging, we sampled the radiodensity of the raw CA images (pixel greyscale value, MATLAB, MathWorks Inc) at ten equally spaced intervals along the length of the main vessel of each phantom type (metal and 3D printed model angiograms). These were compared to a similar number of sample points (n = 90 points in total) from the main vessel of a patient coronary angiogram to enable a comparison. Differences were compared by one-way ANOVA.

### Accuracy of the 3D printing process and reference files

Our validation relied upon the 3D-printed phantom models being a true likeness of the files from which they were printed because these reference files were the gold-standard comparitor. To evaluate this, the phantom models were imaged in a 320-slice computed tomography (CT) scanner (Aquilion Genesis, Toshiba Medical Systems, Japan) at STH. The 3D rendered geometries were extracted using Materialise Mimics software (Materialise, Belgium) and compared with the reference files they were printed from, by calculating the global Hausdorff distance within MeshLab (ISTI-CNR Research Centre, Italy, v2019, https://www.meshlab.net/). This was an important and additional analysis because it ensured our ground-truth comparator i.e. the reference files used to 3D print the phantoms were indeed identical to the phantom models. This analysis (1) validated the accuracy of the 3D printing process and (2) ensured our method of validation (comparing reconstructions agains the print files) was appropriate.

### Validation of the reconstruction method

#### Reproducibility of the method (best achieveable accuracy)

It was important to understand the magnitude of inherent error associated with the comparison method itself, i.e. the highest achievable accuracy. To assess this, we compared each 3D reference file against itself within MeshLab and measured the global Hausdorff distance between the two surfaces. Theoretically, comparing two identical surfaces should result in a Hausdorff distance close to, or equal to, zero. This analysis demonstrated the best achievable Hausdorff distance and the error inherent to the sampling method within the MeshLab software.

#### Accuracy of the minimum lumen diameter

The reconstructed surfaces of the stenosis (metal) phantoms were imported into MeshLab for 3D visualisation. The diameters of non-stenosed and stenosed regions were obtained from the raw centreline data of the reconstructions. These measurements were compared with the phantom minimum lumen diameter (MLD) which was the average of three digital caliper-measured readings at the point of maximum stenosis. 3D reconstructed MLD values were taken as the single smallest radius reading.

#### Accuracy of the luminal surface topography

Each reconstructed surface of the 3D-printed patient-specific models was imported into MeshLab alongside its corresponding reference surface (from which the corresponding phantom model was 3D-printed). The reconstructed and reference geometries were optimally superimposed in 3D space. To ensure maximal overlay and to avoid errors being introduced by imperfect manual positioning, an optimisation script (MATLAB, MathWorks) was used. The similarity between 3D surfaces was quantified and calculating the global Hausdorff distance. The Hausdorff distance objectively quantifies the difference between two geometries by measuring their mutual proximity and measuring the maximal distance between corresponding points of one geometry (reconstructed surface) relative to the other (the reference geometry). We report the minimum, maximum and RMS Hausdorff measurements. This analysis of luminal surface topography also served as a validation of the accuracy of the vessel centreline reconstruction because the latter is dependent upon the former.

#### Assessing the magnitude of error in computed vFFR arising from the 3D CA reconstruction method

The reconstructed and reference surfaces were imported into ANSYS Fluent (ANSYS Inc, Canonsburg, PA) for the generation of a 3D volume mesh to support computational fluid dynamics (CFD) simulation of FFR. Paired samples used identical boundary conditions to ensure a true like-for-like comparison. Boundary conditions represented the coronary microvascular resistance and were based on population data. Virtual FFR (vFFR) was computed using VIRTUheart™ software (University of Sheffield, UK, virtuheart.com, v1.0 (2018)) which derives the translesional pressure ratio from a computational fluid dynamics (CFD) simulation, based upon solving the 3D form of the Navier Stokes equations. Idenitcal boundary condiiotns were used. Thus, any difference in vFFR result must have arisen only from from errors in the 3D CA reconsrtuciton method. Further details of the CFD method are described in detail elsewhere^8^. The computed FFR results for corresponding surfaces were then compared. To support this evaluation all vessels were imaged and reconstructed three times.

### Statistical analysis

3D accuracy of the reconstructed geometries was evaluated by comparing the Hausdorff distances of the reconstructed geometries against those of the reference standard, reported as mean, standard deviation (SD), and 95% confidence interval. The accuracy of the reconstructed stenosis was assessed by comparing the measured and reconstructed stenosis measurements, expressed as distances (mm) and as a percentage of the vessel reference diameter (percentage stenosis). Agreement between the experimental and reference vFFR was assessed by analysing Lin’s concordance correlation coefficient (CCC) which (unlike Pearson’s coefficient) reports covariation *and* correspondence of two variables with the line of correlation passing through zero and a slope of 1.0. Bland–Altman plots were constructed to show differences between vFFR values from reconstructed and reference files. Absolute bias and limits of agreement (± 1.96 SD) were calculated^7^. Statistical analysis was performed using SPSS (v25: IBM Analytics, Armonk, NY, USA).

### Ethical approval

The project was approved by the local and regional ethical review boards.

## Results

### Phantom arterial models

The aluminium stenosis models comprised four eccentric stenoses and three concentric with stenoses ranging from 44.7 to 77.2% representing the mild, moderate and severe clinical range. The seven aluminium and fifteen patient-specific 3D-printed phantom models were each imaged three times, generating 66 analyses in total (Table 1).

**Table 1:** Accuracy of diameter measurement the 3D CA method.

Model	Stenosis	Caliper measured min diameter (mm)	Min diameter from 3D recon (mm)	Delta (mm)	Percentage Diameter stenosis, (caliper) (%)	Percentage Diameter stenosis (recon) (%)	Delta (%)
1	Concentric	0.91	0.90	0.01	71.9	71.5	0.4
2	Concentric	1.43	1.50	− 0.07	55.4	53.1	2.3
3	Concentric	1.65	1.60	0.05	48.6	49.9	1.3
4	Eccentric	1.77	1.84	− 0.07	44.7	44.2	0.5
5	Eccentric	0.88	0.83	0.05	72.8	74.1	1.3
6	Eccentric	0.74	0.72	0.02	77.2	77.5	0.3
7	Eccentric	1.76	1.84	− 0.08	44.7	44.7	0.0

The absolute (mm) and percentage error are reported for each of the seven stenosis models. Percentage stenosis was calculated as the ratio between reference (unstenosed) vessel diameter to diameter at the point of maximum stenosis.

### Radiodensity of the phantoms

The pixel greyscale contrast values of the coronary arteries in the clinical angiograms (n = 30 sample points) were similar to those of the aluminium (n = 30) and 3D-printed (n = 30) phantom models (76.3 ± 15.0 u; 60.0 ± 27.7 u; and 71.7 ± 28.7 u). One-way ANOVA analysis between the samples from each group demonstrated no statistical difference (F (2, 27) = 1.18, P = 0.32). From this we concluded that the phantom model imaging studies were radiographically realistic compared to the clinical imaging studies.

### Accuracy of the phantom models

When comparing the CT imaging results against the reference files, the Hausdorff distance was 0.61 mm (± 0.16 mm, 95% CI 0.45–0.78 mm). We concluded that the printed phantoms were an accurate embodiment of the reference files from which they were printed and, therefore, that the reference files could be used as the ‘gold standard’ reference comparator for the validation analyses.

### Best achieveable accuracy

When the fifteen 3D arterial files were superimposed and compared against themselves (i.e. the same arterial 3D file opended twice and overlayed in 3D space), the Hausdorff distance was 0.28 mm (± 0.20 mm). This defined the best achievable accuracy with this method and the inherent error associated with the sampling method itself. This provided context for assessing the validated accuracy of the reconstruction method.

### Accuracy of the method in reconstructing minimal lumen diameter

For the aluminium stenosis phantom models, compared with the digital caliper measurements, the average error in minimum diameter measurement was 0.05 mm (± 0.03). The error as a percentage of the minimum diameter measurement (i.e. max stenosis) was < 1% (± 0.87%, 95% CI 0.13–1.61%). There was no statistical difference between the caliper measurements made on the phantom models and those from the reconstructions created by the novel method (P = 0.93). Model analysis is detailed in Table 1.

### Accuracy of the method in reconstructing luminal surface topography

**Figure 2:**
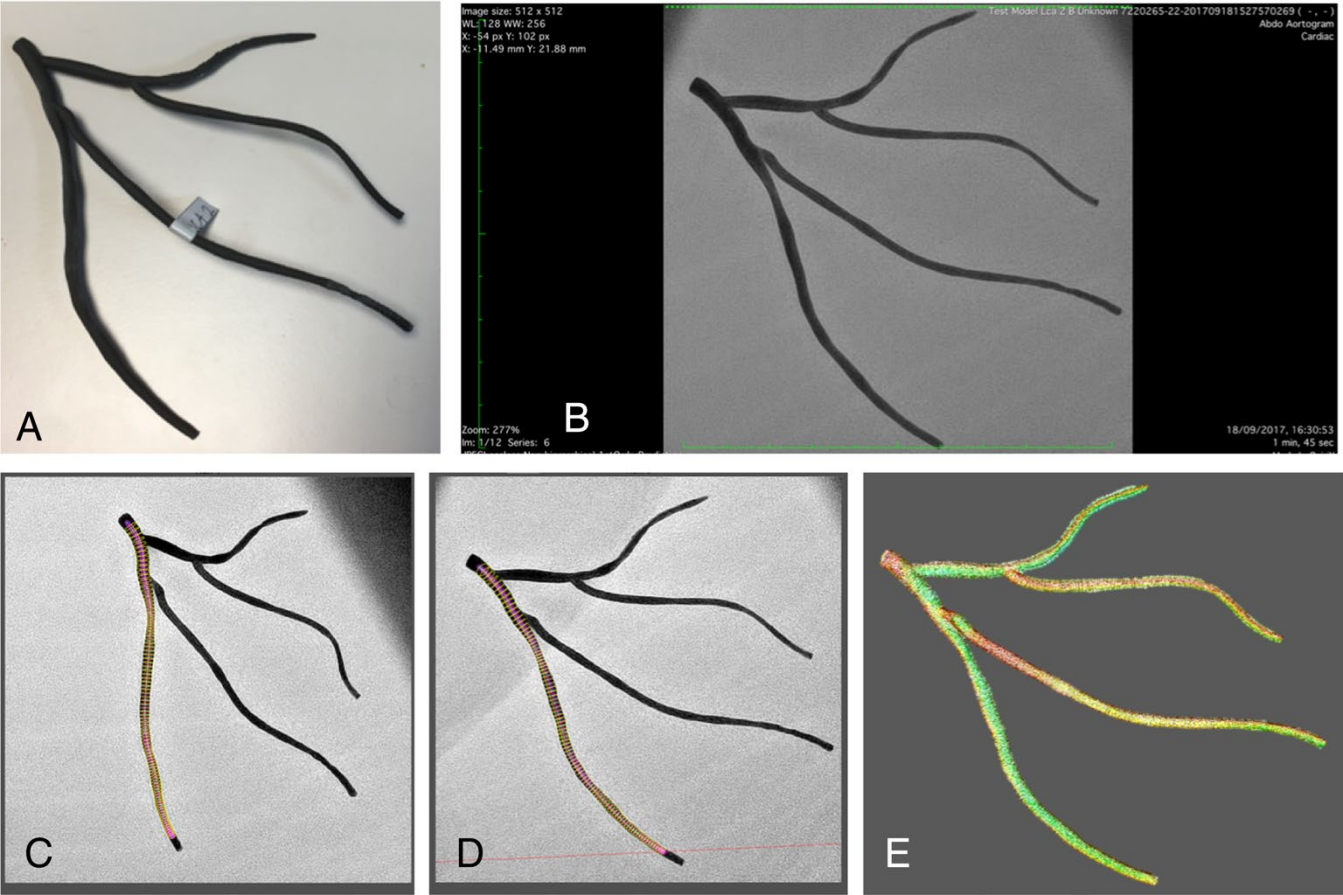
3D-printed left coronary artery phantom (**A**) underwent coronary angiography according to standard clinical protocol in the cardiac catheter laboratory (**B**). Paired images ≥ 30° apart (**C**, **D**) were used to reconstruct the arterial anatomy (**E**). (**E**) is a screen shot from the MeshLab software: the artery reconstructed from imaged phantom is superimposed on top of the artery from the reference file (used to 3D the phantom in **A**) in 3D virtual space. Visual inspection demonstrates excellent agreement but the colour map demonstrates areas of perfect agreement (green) through to areas without perfect correspondence (red).

For all 45 3D CA reconstructions (reconstructed from the 3D-printed phantoms), the Hausdorff distance error, relative to the phantom reference files, was 0.65 mm (± 0.30 mm, 95% CI 0.56–0.74 mm). To help interpret this result, the best achievable Hausdorff distance (from the previous analysis) was 0.28 mm (± 0.20 mm). The following section reports how this error in surface reconstruction translates into error when computing vFFR. Per-vessel accuracy data are presented in Table 2. Figure 2 demonstrates the Hausdorff distance analysis.

**Table 2:** Hausdorff distance analysis of surface similarity, presented on a per-vessel basis for all 45 comparisons.

Model, artery	Minimum (mm)	Maximum (mm)	Mean (mm)	Root mean square (mm)
RCA1 (Main)	0	2.80	0.63	0.84
RCA1 (Main)	0	4.03	0.73	1.01
RCA1 (Main)	0	2.70	0.60	0.83
RCA2 (Main)	0	8.67	1.48	1.94
RCA2 (Main)	0	3.94	0.76	1.00
RCA2 (Main)	0	3.45	0.66	0.86
RCA3 (Main)	0	2.73	0.75	0.98
RCA3 (Main)	0	3.24	0.85	1.10
RCA3 (Main)	0	2.65	0.65	0.82
LCA1 (LAD)	0	3.75	0.61	0.78
LCA1 (LAD)	0	3.12	0.58	0.74
LCA1 (LAD)	0	2.20	0.34	0.44
LCA1 (Diagonal)	0	2.12	0.54	0.68
LCA1 (Diagonal)	0	2.02	0.39	0.52
LCA1 (Diagonal)	0	2.12	0.41	0.55
LCA1 (Marginal)	0	2.69	0.65	0.79
LCA1 (Marginal)	0	3.14	0.77	0.97
LCA1 (Marginal)	0	4.15	0.47	0.67
LCA1 (Circumflex)	0	1.65	0.28	0.36
LCA1 (Circumflex)	0	0.77	0.17	0.22
LCA1 (Circumflex)	0	1.98	0.29	0.37
LCA2 (LAD)	0	2.88	0.44	0.57
LCA2 (LAD)	0	2.02	0.26	0.33
LCA2 (LAD)	0	2.53	0.41	0.54
LCA2 (Diagonal)	0	2.19	0.38	0.49
LCA2 (Diagonal)	0	2.70	0.40	0.45
LCA2 (Diagonal)	0	1.48	0.32	0.41
LCA2 (Marginal)	0	1.74	0.24	0.30
LCA2 (Marginal)	0	1.84	0.22	0.30
LCA2 (Marginal)	0	2.65	0.59	0.75
LCA2 (Circumflex)	0	1.82	0.29	0.35
LCA2 (Circumflex)	0	2.95	0.30	0.43
LCA2 (Circumflex)	0	1.80	0.28	0.34
LCA3 (LAD)	0	2.01	0.44	0.57
LCA3 (LAD)	0	2.88	0.45	0.61
LCA3 (LAD)	0	1.58	0.35	0.46
LCA3 (Diagonal)	0	3.00	0.63	0.88
LCA3 (Diagonal)	0	2.63	0.59	0.77
LCA3 (Diagonal)	0	1.44	0.37	0.46
LCA3 (Marginal)	0	1.99	0.35	0.48
LCA3 (Marginal)	0	2.02	0.59	0.71
LCA3 (Marginal)	0	1.95	0.59	0.72
LCA3 (Circumflex)	0	4.04	0.44	0.65
LCA3 (Circumflex)	0	2.02	0.59	0.71
LCA3 (Circumflex)	0	1.63	0.30	0.37

Measurements include minimum, maximum and mean distances between sampled points on reference and reconstructed 3D vessels when aligned and superimposed. Root mean square is the most important result, providing an overall value of error in reconstruction of each vessel in millimetres.

### Accuracy of the 3D CA reconstruction method when computing vFFR

**Figure 3:**
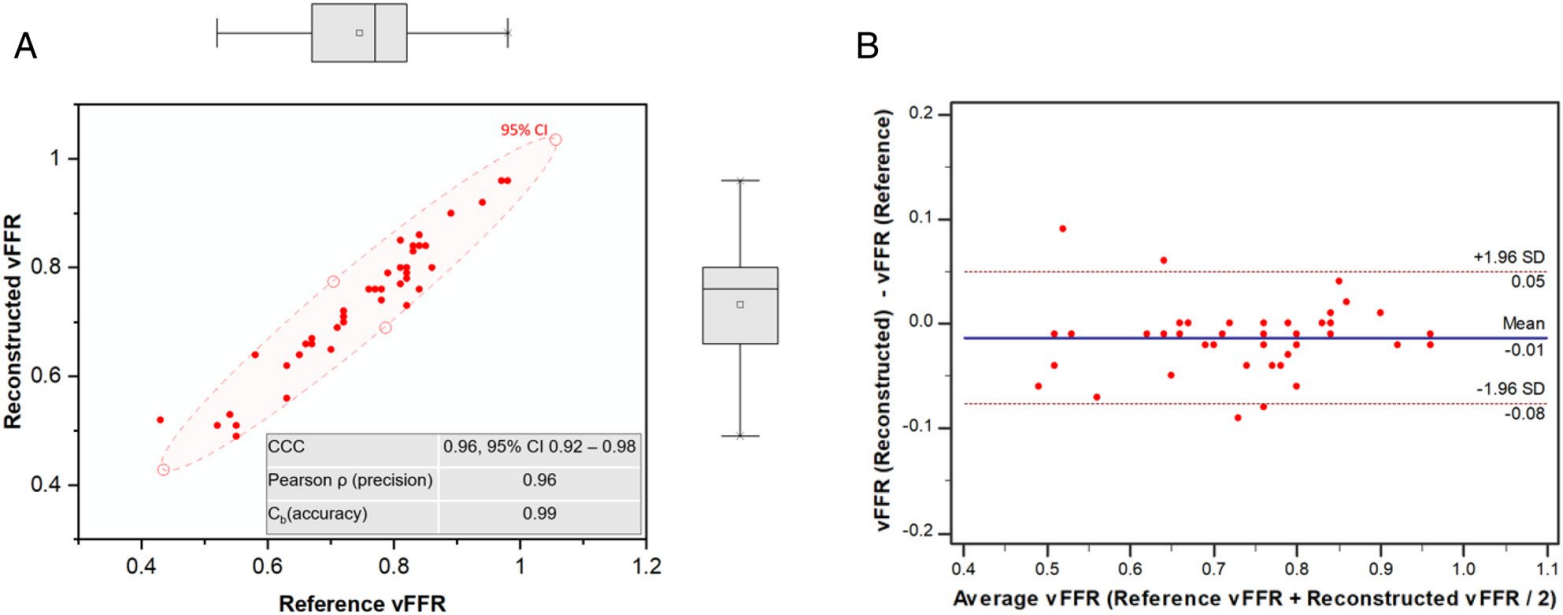
(**A**) Scatter plot demonstrating high concordance correlation coefficient (CCC) between between angiography-derived vFFR from the 3D reconstruction method and the gold-standard reference file-derived vFFR. (**B**) Bland–Altman Plot demonstrating agreement and differences between angiography-derived vFFR from the 3D CA reconstruction method and the gold-standard reference file-derived vFFR. Mean delta (bias) is shown as a solid line (− 0.01) and the upper and lower limits of agreement (SD: ± 1.96) are shown with the interrupted line (− 0.07 to + 0.05).

**Figure 4:**
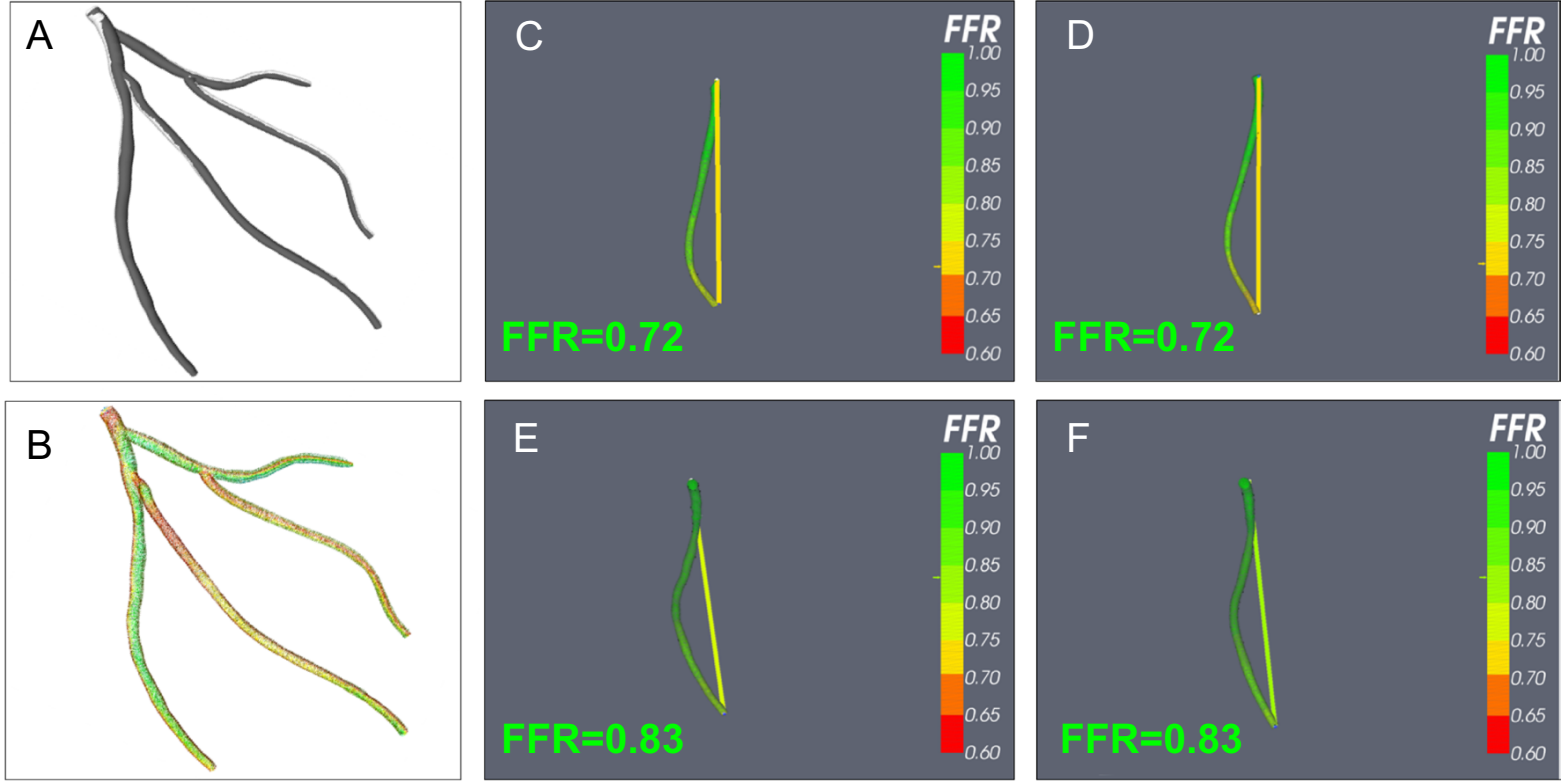
vFFR analysis. Individual braches of the reference file (**A**) and the 3D CA reconstructed artery (**B**) were subjected to vFFR analysis under identical conditions. (**C**, **D**) Show the obtuse marginal branch from the reference file (**C**) and 3D CA (**D**). Excellent anatomical reconstruction means that both fluid dynamics analyses result in vFFR of 0.72. (**E**, **F**) Demonstrate the analysis for the left anterior descending branch. In this branch both the reference and 3D CA reconstructed vessles have a vFFR of 0.83*.*

The mean vFFR of the 45 reference vessels was 0.75 (± 0.12) and the range was 0.43–0.98. Twenty-five cases (56%) were below the threshold value for physiological significance (≤0.80) and twenty-one (47%) were within the zone 0.75–0.85. Mean vFFR of the vessels reconstructed from the 3D-printed phantom models (n = 45) was 0.73 (± 0.12) and the range was 0.49–0.96. Relative to the reference vessels, the reconstructions had a small but statistically significant negative bias in vFFR (mean delta − 0.01 ± 0.03, P < 0.05) (Fig. 3). In addition, the concordance correlation coefficient between reference and 3D CA reconstructed vFFR results was excellent (CCC = 0.96, 95% CI 0.92–0.98) (Fig. 3). Case-specific comparison of the reconstructed and reference vessels is displayed in Table 3. Figure 4 demonstrates an example of vFFR analysis of the reconstructed and reference surfaces. Relative to the vFFR results computed from the reference surfaces, the Bland Altman 95% limits of agreement of the vFFR results computed from the reconstructed surfaces were ± 0.06.

**Table 3:** Per-vessel vFFR measurements for reconstructed and reference meshes.

Vessel	Model name	vFFR (print)	vFFR (recon)	Absolute error (delta vFFR)	Bias (delta vFFR)
LAD	LCA1A	0.76	0.76	0.00	0.00
Diagonal	LCA1A	0.72	0.71	0.01	− 0.01
Marginal	LCA1A	0.82	0.73	0.09	− 0.09
Circumflex	LCA1A	0.72	0.70	0.02	− 0.02
LAD	LCA2A	0.84	0.84	0.00	0.00
Diagonal	LCA2A	0.86	0.80	0.06	− 0.06
Marginal	LCA2A	0.70	0.65	0.05	− 0.05
Circumflex	LCA2A	0.63	0.62	0.01	− 0.01
LAD	LCA3A	0.67	0.66	0.01	− 0.01
Diagonal	LCA3A	0.65	0.64	0.01	− 0.01
Marginal	LCA3A	0.79	0.79	0.00	0.00
Circumflex	LCA3A	0.52	0.51	0.01	− 0.01
Main	RCA1A	0.81	0.85	0.04	0.04
Main	RCA2A	0.55	0.51	0.04	− 0.04
Main	RCA3A	0.82	0.79	0.03	− 0.03
LAD	LCA1B	0.83	0.84	0.01	0.01
Diagonal	LCA1B	0.89	0.90	0.01	0.01
Marginal	LCA1B	0.98	0.96	0.02	− 0.02
Circumflex	LCA1B	0.77	0.76	0.01	− 0.01
LAD	LCA2B	0.83	0.83	0.00	0.00
Diagonal	LCA2B	0.97	0.96	0.01	− 0.01
Marginal	LCA2B	0.72	0.72	0.00	0.00
Circumflex	LCA2B	0.76	0.76	0.00	0.00
LAD	LCA3B	0.76	0.76	0.00	0.00
Diagonal	LCA3B	0.94	0.92	0.02	− 0.02
Marginal	LCA3B	0.79	0.79	0.00	0.00
Circumflex	LCA3B	0.67	0.67	0.00	0.00
Main	RCA1B	0.85	0.84	0.01	− 0.01
Main	RCA2B	0.58	0.64	0.06	0.06
Main	RCA3B	0.84	0.86	0.02	0.02
LAD	LCA1C	0.71	0.69	0.02	− 0.02
Diagonal	LCA1C	0.65	0.64	0.01	− 0.01
Marginal	LCA1C	0.82	0.80	0.02	− 0.02
Circumflex	LCA1C	0.78	0.76	0.02	− 0.02
LAD	LCA2C	0.81	0.80	0.01	− 0.01
Diagonal	LCA2C	0.78	0.74	0.04	− 0.04
Marginal	LCA2C	0.81	0.77	0.04	− 0.04
Circumflex	LCA2C	0.63	0.56	0.07	− 0.07
LAD	LCA3C	0.67	0.66	0.01	− 0.01
Diagonal	LCA3C	0.66	0.66	0.00	0.00
Marginal	LCA3C	0.43	0.52	0.09	0.09
Circumflex	LCA3C	0.54	0.53	0.01	− 0.01
Main	RCA1C	0.84	0.76	0.08	− 0.08
Main	RCA2C	0.55	0.49	0.06	− 0.06
Main	RCA3C	0.82	0.78	0.04	− 0.04

The observed error in physiological simulation for 3D reconstructed vessels when compared to their reference (print mesh) counterparts is also reported.

## Discussion

We have developed and validated a method for reconstructing 3D coronary artery anatomy from standard CA suitable for using as the basis for the computation of vFFR. The method uses a simple protocol of two standard 2D angiographic image projections separated by at least 30°. The method compensates for inter-acquisition patient and X-ray table movement in any plane, and for intra-acquisition movement or ‘panning’. Using both idealised metallic, and patient-specific 3D-printed phantom coronary models, we have demonstrated good geometric accuracy of the reconstruction method in terms of both minimum lumen diameter capture and overall surface similarity. The reconstruction was sufficient for reliable physiological simulation (computing vFFR). vFFR agreement with the reference results was also good. Relative to the results computed from the reference surfaces, the 95% Bland Altman limits of agreement of the vFFR results computed from the reconstructed surfaces were ± 0.06 and the concordance correlation coefficient was 0.96.

Reconstructing 3D coronary anatomy from conventional (2D) CA is challenging. Epicardial coronary arteries are only 2–5 mm in diameter, smaller in stenosed regions, tortuous, and constantly moving due to cardiac, ventilatory and patient activity. In addition, the X-ray table is moved between and during image acquisitions, arteries overlie each other, and an individual projection may not adequately show all regions of interest. Early innovations attempted to overcome some of these challenges by rotating the C-arm while acquiring images, or by simultaneous biplane acquisition; but these systems are not widely available, are cumbersome, and associated with significant practical shortcomings^13,14^. A strength of the methods described this study is that a standard multiple single-plane CA is all that is required to reconstruct the coronary anatomy. 3D CA reconstruction was originally developed for 3D qualitative coronary angiography (3D-QCA) 42^15^ which is superior to 2D methods for assessing eccentricity and length 44^1^, visualising bifurcation anatomy^16^, and predicting flow limitation^2,17^.

Uniquely, but importantly, our study studied the association between *geometric* and computed *physiological* error. The physiological (vFFR) error arising purely from errors in the reconstruction was FFR ± 0.06 (Bland–Altman 95% limits of agreement). Whilst this is clinically relevant, it needs placing in context. A recent meta-analysis of thirteen studies of vFFR reported average error of vFFR ± 0.14^11^. The previously reported error of the current vFFR method was FFR ± 0.16^8^. Similarly, a study of vFFR computed from CT angiography reported an error of ± 0.15 (24)^18^. Thus, our data suggest that errors in the reconstruction process do account for a significant proportion of vFFR error. This corresponds with the findings of published vFFR sensitivity analyses which highlight that the major sources of vFFR error arise from the selection and tuning of boundary conditions to represent the microvascular resistance, followed by the anatomical reconstruction^7,19,20^. Thus, the present study supports the notion that, in the context of the computation of vFFR from coronary arterial reconstructions, the accuracy of the reconstruction process is an important determinant of vFFR accuracy.

In the context of the MLD, which is important when computing vFFR, our method was associated with an overall < 1% error compared with digital caliper measurement. In terms of surface similarity, the lowest possible achievable error (when we compared identical surfaces) was 0.28 mm (± 0.20 mm). The error of our reconstruction method (0.65 mm ± 0.30 mm) was therefore only 0.37 mm above the best achievable. Given that the pixel sizing of the Philips angiography system was 1 pixel = 0.314 mm (at 512 × 512 pixel resolution), our model was accurate, at worst, to two pixels; and, at best, to a single pixel. This is reassuring because no system can be more accurate than a single pixel. It would therefore be interesting to test the method on higher resolution detectors (1024 × 1024)^21^. Furthermore, the error we observed in our method was almost identical to that of high-resolution CT reconstruction (0.65 mm ± 0.30 novel vs 0.61 mm, ± 0.16 CT), an imaging modality used routinely in clinical practice for coronary angiographic reconstructions and vFFR computation^22^.

Our analysis was more detailed and pragmatic than previously published studies. Shechter et al.^23^, Movassaghi et al.^24^, and Yang et al.^25^ all used physical coronary phantom models to assess the accuracy of their 3D arterial reconstructions. However, these studies considered only the accuracy of reconstructed centreline data, using Euclidian distance measurement^23–25^. This is different to our study because we also integrated an assessment of the luminal surface topography (which incorporates centreline accuracy), stenosis capture, and physiological accuracy. Furthermore, the models used in these studies were relatively simple, contrast-filled tubes with narrowings made to mimic stenoses, not reflecting actual patient anatomy. One of the challenges of reconstructing 3D models from 2D angiographic images is in capturing vessel curvature and dealing with images that foreshorten the arterial anatomy. It would not be possible to assess this in these phantom types. In their comparison of centreline data, Yang et al., described their average positional accuracy (distance between reconstructed and true phantom centrelines) to be 0.665 mm, similar to the average error in surface reconstruction reported in our study^25^. Centreline comparison provides an analysis of the system’s ability to capture the curvature of the vessel, however it gives no information on the quality of surface topography or diameter accuracy. Shechter et al. subjected their phantoms to magnetic resonance imaging and used the resultant reconstructed centreline data as their comparator^23^. Arguably, using this method, errors in the process of obtaining centreline data for the imaged phantoms may influence the results; it is more a comparison of reconstructions rather than validation against a ground truth comparator.

Other methods of validation have been used. In an in vivo assessment of the accuracy of its 3D vessel reconstruction, the Cardiovascular Angiographic Analysis Systems (CAAS) QCA-3D (Pie Medical Imaging, BV, NL), ten patients underwent coronary angiography and intravascular ultrasound (IVUS). Reconstructed arterial anatomy was compared with the IVUS data^26^. Although the CAAS QCA-3D system underestimated luminal area and lesion length, it did show a strong correlation with IVUS derived dimensions. IVUS itself has multiple drawbacks in vessel analysis: probe placement and choice of imaging plane can introduce geometrical inaccuracies, image quality is low and most importantly in this case, it produces a straight tube and cannot represent the 3D shape or curvature of vessels (22)^27^. The CAAS workstation is now providing commercially available vFFR simulation, using 3D arterial anatomy.

The physiological significance of the cases included in this study was clinically relevant. Sixty percent were physiologically significant and nearly half (47%) were in the borderline range (0.75–0.85). One of the greatest challenges for reconstructing coronary anatomy from angiography, particularly when computing physiology, is the ability to accurately capture small diameters at the point of maximum stenosis (minimum vessel diameter)^28^. Small changes in diameter have a relatively large influence on CFD analysis of computed pressure gradient and vFFR. A strength of the current study was the use of clinically realistic stenotic phantoms. The phantoms comprised of curved and linear geometries, concentric and eccentric stenoses and minimum diameters ranging from 0.74 to 1.77 mm (45–77%). These combinations reflect real-world CAD, investigated and treated in the cardiac catheter laboratory within the anatomically mild, moderate and significant range^29^. Despite the tightest stenosis being just over two pixels wide on angiography (0.74 mm), our tool captured this to within 0.02 mm of error. This same value also defined the average error across all stenosis analyses. Most X-ray angiography-based modelling tools generate axisymmetric reconstructions based upon average radius calculation. Although this is ideal for concentric lesions, it is not representative of real-world atherosclerotic plaque that is usually eccentric, at least to a small degree^30^. It is, therefore, possible that most tools for this type of 3D reconstruction are introducing a small magnitude of error as a result of circular diameter approximation. Galassi et al. refined their 3D reconstruction algorithm to better account for more complex luminal contours by using a Non-uniform Rational B Spline contour, allowing for flexible freeform diameter reconstruction^21^. A comparison of luminal diameter was made against intravascular imaging using optical coherence tomography. In a sample of vessels with true diameters ranging between 0.72 and 1.03 mm, the average error was 0.29 mm despite the use of ‘flexible’ luminal modelling. Our method still supports the analysis of eccentric stenoses, including those in the current study, because the centreline deviates accordingly. Nevertheless, this is a potential limitation, and there are algorithms that compute non-circular cross-sections: we would observe that there is simply not enough information in two projections to identify the shape of the cross-section uniquely, although the more sophisticated processes certainly produce better approximations than axisymmetric^21^. However, the primary purpose of this process was to produce geometries to support physiological computation, and separate exploration of the sensitivity of this parameter to the assumption of circular cross-sections had indicated that this is a minor limitation in comparison to other sources of error, especially estimation of the distal myocardial resistance^19^.

The sample size in our study was small. However, it was larger than those used in other validation studies in this area. It also utilised patient-specific 3D-printed models^23–25^. This enabled a unique, clinically relevant analysis. Finally, although our phantom coronary models are based on real patient anatomies, no phantom is a perfect representation of reality. Additional movement due to the cardiac and respiratory cycles were not captured. The results of this analysis are therefore, informative and hypothesis-generating, and may underestimate the true magnitude of error arising from the reconstruction process, compared with real-world clinical use.

## Conclusions

We have developed and validated a tool that reconstructs 3D coronary arterial anatomy from standard CA. Using phantom coronary models, the method was simple to use and compensated effectively for inter- and intra-acquisition movement. Errors in reconstruction resulted in small but clinically relevant errors in the computation of vFFR. Although derived from clinically realistic arterial phantom models, these errors may underestimate those experienced in real-world clinical datasets due to additional movements associated with the cardiac and respiratory cycles, not fully captured in this study.

## Supplementary Information

Supplementary Tables.

## Abbreviations

2D and 3D: Two and three-dimensional
CA: Coronary angiography
CAD: Coronary artery disease
CFD: Computational fluid dynamics
CT: Computed tomography
FFR: Fractional flow reserve
HU: Hounsfield units
IVUS: Intravascular ultrasound
LCA: Left coronary artery
LAD: Left anterior descending
LCX: Left circumflex
PCI: Percutaneous coronary intervention
RCA: Right coronary artery
SD: Standard deviation
STH: Sheffield Teaching Hospitals
QCA: Quantitative coronary angiography
vFFR: Virtual fractional flow reserve
