# Supplementary material for: The importance of three dimensional coronary artery reconstruction accuracy when computing virtual fractional flow reserve from invasive angiography
Source: Sci Rep. 2021 Oct 4;11:19694. doi: 10.1038/s41598-021-99065-7 (PMC8490364; doi:10.1038/s41598-021-99065-7)
Supplement: Supplementary file 1 — Supplementary Tables. [file 41598_2021_99065_MOESM1_ESM.docx]

**Supplementary Material**

**The importance of three dimensional coronary artery reconstruction accuracy when computing virtual fractional flow reserve from invasive angiography.**

**Solanki et al. (SREP: 3b12a948-aac4-41f1-a2e6-7401903feebb)**

**Supplementary Table 1**

| **Model** | **Phantom Type** | **Stenosis Type** | **Lesion length (mm)** | **Average Maximum Stenosis Diameter (mm)** | **Percentage Stenosis (%)** |
| --- | --- | --- | --- | --- | --- |
| **1** | Straight | Concentric | 10 | 0.91 | 71.9 |
| **2** | Straight | Concentric | 12 | 1.43 | 55.4 |
| **3** | Straight | Concentric | 9 | 1.65 | 48.6 |
| **4** | Straight | Eccentric | 15 | 1.77 | 44.7 |
| **5** | Straight | Eccentric | 13 | 0.88 | 72.8 |
| **6** | Curved | Eccentric | 18 | 0.73 | 77.2 |
| **7** | Curved | Eccentric | 20 | 1.76 | 44.7 |

**Table S1.** Lesion parameters for fabricated stenosis on metallic phantom models

**Supplementary Table 2**

| **Artery** | **Optimal projections** |
| --- | --- |
| Left Main Stem | LAO-caudal and LAO-cranial |
| Left Anterior Descending (LAD) | PA-caudal and RAO-caudal for proximal vessel and RAO-cranial, PA-cranial and LAO cranial for mid to distal vessel |
| Left Circumflex | RAO-caudal and LAO-caudal |
| Right Coronary Artery (RCA) | Straight LAO, straight RAO for proximal vessel and PA-cranial for distal |

**Table S2.** Projection positions for imaging particular branches of coronary arterial vasculature. LAO: left anterior oblique; RAO: right anterior oblique; PA: postero-anterior*.*
